# EasyGO: Gene Ontology-based annotation and functional enrichment analysis tool for agronomical species

**DOI:** 10.1186/1471-2164-8-246

**Published:** 2007-07-24

**Authors:** Xin Zhou, Zhen Su

**Affiliations:** 1 Division of Bioinformatics, State Key Laboratory of Plant Physiology and Biochemistry, College of Biological Sciences, China Agricultural University, Beijing 100094, China

## Abstract

**Background:**

It is always difficult to interpret microarray results. Recently, a handful of tools have been developed to meet this need, but almost none of them were designed to support agronomical species.

**Description:**

This paper presents EasyGO, a web server to perform Gene Ontology based functional interpretation on groups of genes or GeneChip probe sets. EasyGO makes a special contribution to the agronomical research community by supporting Affymetrix GeneChips of both crops and farm animals and by providing stronger capabilities for results visualization and user interaction. Currently it supports 11 agronomical plants, 3 farm animals, and the model plant *Arabidopsis*. The authors demonstrated EasyGO's ability to uncover hidden knowledge by analyzing a group of probe sets with similar expression profiles.

**Conclusion:**

EasyGO is a good tool for helping biologists and agricultural scientists to discover enriched biological knowledge that can provide solutions or suggestions for original problems. It is freely available to all users at .

## Background

High-throughput technologies such as microarray techniques can study thousands of biological entities simultaneously. Extracting the important biological facts from the results of such experiments is of crucial importance, but has proven difficult for experimental biologists. To solve this problem, a systemized annotation vocabulary describing biological knowledge and tools to uncover hidden knowledge automatically using such a vocabulary are required. The Gene Ontology (GO) annotation system [1] can meet this requirement by providing a set of expert-curated terms describing biological entities in three aspects (biological process, molecular function, and cellular component) organized into a hierarchical structure. Genes and microarray probe sets could be associated with certain GO terms according to the biological functions they perform or represent, and enriched terms in a GO-annotated list of genes or probe sets could be used to characterize biological "theme" in the list. Many software and web servers have been developed for this purpose and are summarized in a recent paper [2]. However, from the vantage point of the agronomical research community, almost no tools have been designed to support agronomic species like crops and farm animals, except for a few model organisms [3-10]. In addition, many current tools display analysis results in the form of tables or ranked lists [5-9,11], which is uninformative to users as Gene Ontology is hierarchical in nature.

This paper presents EasyGO, a web-based tool to perform GO-based functional enrichment analysis for crop and farm animal species, including Affymetrix GeneChips for 12 plants and 3 farm animals, together with *Arabidopsis *and rice (*indica *and *japonica*) gene names. The annotation data for all GeneChip probe sets were regenerated by the best BLAST hit method to obtain better annotation coverage than that available from manufacturer-provided data in a reasonable way, thus making EasyGO's service more informative. In the form of statistically enriched terms, analysis results are visualized within the rich structure of a GO hierarchical tree, thus becoming much comprehensible. By focusing on the above points, EasyGO is expected to be more suitable than other currently available tools for the needs of the agronomical research community.

## Construction and content

### Implementation

EasyGO is a web-based tool, so that no software installation effort is required. It is composed of two parts: a MySQL database containing GO annotation data for supported data types, and server-side Perl scripts for functional enrichment analysis and results display. The R software [12] is used to process statistical tests, and the dot program of the Graphviz software [13] is used to generate directed acyclic graphs.

### Generation of GO annotation data

Currently, EasyGO supports Affymetrix GeneChips for 12 plant species (*Arabidopsis*, rice, wheat, maize, barley, sugar cane, soybean, poplar, medicago, citrus, cotton, and tomato) and 3 animal species (chicken, bovine, and porcine). We regenerated GO annotation for the GeneChip probe sets to obtain better annotation coverage than could be achieved using manufacturer-provided data (comparison of annotation coverage between the two sources of data is available online as additional file 1). For this purpose, the best BLAST hit method [14] was used to transfer GO annotation from the annotated sequence to the unannotated sequence if the annotated sequence is the BLAST top hit of the unannotated sequence under a certain E-value cutoff. Gene product GO annotations are available on the Gene Ontology Consortium website for some of the above species (*Arabidopsis*, rice, chicken, bovine, and UniProt multi-species GO annotations) and were downloaded in November 2006. Meanwhile, gene product sequences were retrieved from public sequence databases (TAIR, UniProt, Ensembl, and GenBank). These data were used to construct BLAST databases for annotating GeneChip probe sets. Consensus or exemplar sequences of GeneChip probe sets were blasted against corresponding sequence databases, and top hits were selected using an E-value cutoff of 10^-30^. Probe sets failed to obtain top hits were re-blasted against a sequence database with wider scope, and the same E-value cutoff was used to select top hits for them, so that more probe sets could be annotated. BLAST database selection and annotation status for all GeneChips is available online as Additional file 1.

### Functional enrichment analysis

In EasyGO, functional enrichment analysis is done by finding GO terms with unbalanced distribution between two groups of genes or probe sets. By default, EasyGO compares a query list with a previously computed background composed of all known genes for a species or all probe sets on a GeneChip. In practice, user can submit a customized reference list when the default background is inappropriate (e.g., if it is desired to use expressed probe sets as a background, while only a portion of probe sets are expressed in a typical expression profiling experiment). Mapping count, which is the number of list entries annotated by the term, is calculated for each term in both lists, and the "true path rule" is applied so that each list entry contributes not only to the mapping counts of the terms assigned to it, but also to all parental terms on paths to the root term. The mapping counts are used to calculate each term's enrichment level in the query list, for which purpose three statistical test methods (binomial, *χ*^2^, and hypergeometric tests) can be used in EasyGO. In the binomial test, the annotation status of each query entry (whether or not the entry is annotated by a certain term) is regarded as a Bernoulli trial, and its probability of being annotated equals the frequency of annotated entries in the background (reference) list, thus the P-value of generating annotation status from the query list can be calculated from the resulting binomial distribution. In the *χ*^2 ^test, term mapping counts in the query and background (reference) lists are used to form a 2 × 2 contingency table, from which the difference between observation and expectation for each category is measured to derive a P-value from a *χ*^2 ^distribution with one degree of freedom. The hypergeometric test uses the hypergeometric distribution to calculate the probability of obtaining the contingency table as created above by chance. When the input list is compared with the previously computed background, or when it comprises a subset of the reference list, the enrichment problem is best modeled by the hypergeometric distribution. When the input list has few or no intersections with the reference list, the binomial and *χ*^2 ^tests are more appropriate. In consideration of multiple testing issues, a false discovery rate (FDR) correction [15] is performed on the P-values to control falsely rejected hypotheses.

## Utility and discussion

### Web interface and results display

**Figure 1:**
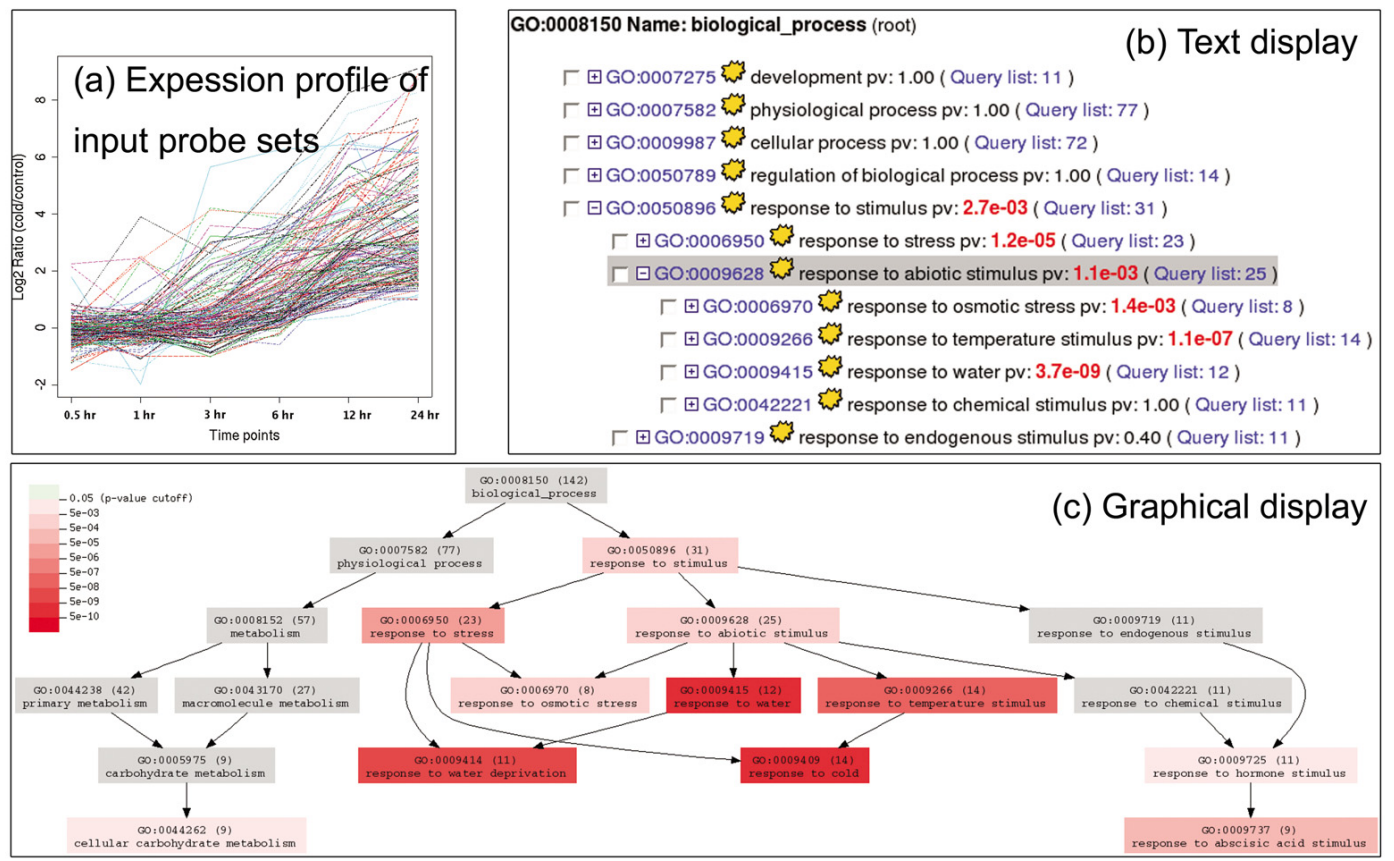
**Results of the case study**. Figure 1a shows the cold-induced expression of the list of probe sets used in the case study; their expression change show a common trend during the six time points of the experiment. Figure 1b and 1c show analysis results of the case study in text and graphical forms.

Analysis using EasyGO starts from a simple interface where user options are presented. The analysis results are returned in either text or graphic mode. In text mode (Figure. 1b), a text-based tree is produced to show term relationships. Each term occupies a row, and information like the FDR-corrected P-value and its mapping count is displayed. Children terms can be expanded or collapsed by clicking on the parental term. In graphic mode (Figure. 1c), term relationships are visualized using a tree-like graph where nodes are terms and their background colors are determined by the corrected P-values. Both display modes enable the user to access the set of genes or probe sets annotated by a term of interest and view detailed information for that term including annotation and BLAST top hit information.

### Case study

To demonstrate the use of EasyGO, a case study is performed using 168 *Arabidopsis *ATH1 GeneChip probe sets. The probe sets show coordinated expression level in shoot tissue under cold treatment, and a plot of their expression levels can be seen in Figure. 1a. By default, all non-control probe sets were used as a background, and analysis was performed on the aspect of "biological process" using the hypergeometric test. From the results display shown in Figures. 1b and 1c, terms associated with the stress-response property are enriched in the query list, for example GO:0009266 (response to temperature stimulus) and GO:0006970 (response to osmotic stress). This indicates that the query list contained plenty of cold- and water stress-responsive genes, which agrees with current findings that cold- and water stress-response mechanisms have cross-talk in *Arabidopsis *[16].

### Future improvements

Currently, the GO annotation data stored in EasyGO are derived from sequence similarity search. With this approach, the actual similarity between query sequence and top hit sequence may be of concern when judging the reliability of transferred GO annotation. To address this concern, the authors are planning to weight transferred GO annotation using the percentage of the sequence region conserved between query and top hit. Also, a suitable test method that operates with a weighted mapping count needs to be developed. In addition, to make EasyGO more widely applicable, the authors intend to include expression microarrays for agronomical species from other companies such as Agilent and Operon.

## Conclusion

The research reported here has given researchers studying the molecular biology of crops and farm animals a new tool to interpret high-throughput experimental results, such as a list of probe sets from expression microarrays. As described above, minimum user effort is required to use EasyGO, and its analysis results are displayed in an easy-to-read style. The authors believe that in practice, EasyGO will meet the general requirements of users in this field and facilitate their research work.

## Availability and requirements

EasyGO is freely available for all users at .

## Authors' contributions

XZ performed GO annotation and database and web server construction and compiled the first draft of the manuscript. ZS supervised the project. Both authors read and approved the final manuscript.

## Supplementary Material

Additional file 1 BLAST database selection and annotation status for each supported data type. Click here for file
